# A STUDY ON THE TYPES OF CHANGES IN DEPRESSION AND COVID-19 AMONG OLDER ADULTS IN KOREA

**DOI:** 10.1093/geroni/igad104.2143

**Published:** 2023-12-21

**Authors:** Kyu-Hyoung Jeong, Ju Hyun Ryu, Seoyoon Lee, Sunghee Kim

**Affiliations:** Semyung University, Jecheon, Republic of Korea; Yonsei University, Seoul, Republic of Korea; Yonsei University, Seoul, Republic of Korea; Yonsei University, Seoul, Republic of Korea

## Abstract

This study focuses on the long-term trends in depression levels in older adults, with a particular focus on the period following the onset of COVID-19. While previous studies have examined depression levels in older adults, this study aims to complement those studies by providing a longitudinal perspective. This study used data from the Korea Welfare Panel Study(KoWePS), which included 2,716 data of older adults aged 65 and above, from 2017 to 2021. The study variables included gender, age, income, educational background, residential area, living alone, and disability status. The results of this study identified two types of changes in depression levels in older adults: the ’rapidly rising’ type and the ’steadily increasing’ type. The study found that those who were more likely to belong to the ’rapidly rising’ type were those with lower equalized annual income, women, those with lower education, those living in urban areas, and those living alone. These findings suggest that those who are already at risk for depression are at even higher risk during and after the COVID-19 pandemic. Given these findings, it is crucial to provide timely and effective guidelines to prevent depression in older adults during outbreaks of infectious diseases such as COVID-19. Policymakers should consider the findings of this study when implementing policies aimed at preventing depression in older adults. Overall, this study provides important insights into the long-term trends in depression levels in older adults and highlights the importance of addressing this issue in response to the ongoing COVID-19 pandemic.

